# Comparative Efficacy of Carbonyl Sulfide Against Phosphine-Resistant and Phosphine-Susceptible Strains of the Rice Weevil *Sitophilus oryzae*

**DOI:** 10.3390/insects17030347

**Published:** 2026-03-21

**Authors:** Bong-Su Kim, Ji-Eun Choi, Jae-Ho Ban, Soo-Jung Suh, Jun-Ran Kim

**Affiliations:** Department of Plant Quarantine, Animal and Plant Quarantine Agency (APQA), Gimcheon 39660, Republic of Korea; bskim79@korea.kr (B.-S.K.);

**Keywords:** phosphine resistance, fumigation, carbonyl sulfide, *Sitophilus oryzae*

## Abstract

Recently, the occurrence of phosphine-resistant pests has been increasingly reported in many countries. In this study, the efficacy of carbonyl sulfide (COS) on phosphine-resistant and phosphine-susceptible strains of the rice weevil, *Sitophilus oryzae*, was evaluated to determine the applicability of COS as a fumigant to control phosphine resistance. *S. oryzae* at the egg, larval and adult stages was treated with phosphine and COS to determine the 50 and 99% lethal concentration time (LCt_50_ and LCt_99_, respectively) values. The LCt_50_ values of phosphine for phosphine-susceptible *S. oryzae* at the egg, larval and adult stages were 1.44, 0.63, and 0.66 mg h/L, respectively, and those for phosphine-resistant *S. oryzae* were 30.65, 17.60, and 8.37 mg h/L, respectively. In contrast, the LCt_50_ values of COS for phosphine-susceptible *S. oryzae* at the egg, larval, and adult stages were 284.19, 171.11 and 212.55 mg h/L, respectively, and those for phosphine-resistant *S. oryzae* were 289.78, 149.87 and 229.06 mg h/L, respectively. The COS-resistance ratios were 1.02, 0.88, and 1.08 for *S. oryzae* at the egg, larval, and adult stages, respectively. These results indicate that the efficacy of COS is similar for phosphine-susceptible and phosphine-resistant pests, suggesting that COS can be used to control phosphine-resistant grain pests.

## Simple Summary

Phosphine resistance has become a serious problem worldwide. This study was conducted to evaluate the feasibility of using a new fumigant, carbonyl sulfide (COS), to control the rice weevil (*Sitophilus oryzae*), a pest known to be resistant to phosphine. Tests were performed to treat phosphine-resistant and phosphine-susceptible rice weevils with either phosphine or COS to derive concentration time (Ct) values, analyze the control effects, and compare the resistance ratios. The test results revealed that compared with phosphine-susceptible rice weevils, phosphine-resistant rice weevils exhibited more than 20-fold greater resistance to phosphine. However, the resistance ratio to COS was close to 1, indicating no difference in efficacy between resistant and susceptible pests. These results suggest that COS may be applicable to control phosphine-resistant pests. However, further research, such as combined treatment, is needed to increase its field applicability.

## 1. Introduction

Pest control during grain storage is essential for maintaining the quantity and quality of grain. Grain pests, such as *Sitophilus oryzae*, *Tribolium castaneum*, *Rhyzopertha dominica* and *Oryzaephilus surinamensis*, are known to cause significant damage to stored grain [1]. In India, *S. oryzae* causes damage to 10 to 65% of grains under moderate storage conditions [2]. In Ethiopia, 14% of wheat losses are reported to be caused by stored grain pests, including *Sitophilus* spp. [3]. Moreover, grain pests can affect the quality of grain [4]. Several pest control methods, such as chemical spray and cold and heat treatments, are available, and the most commonly used method to control pests during grain storage is phosphine fumigation [5]. Phosphine has long been used as a plant quarantine and agricultural fumigant to control insect pests, and it is known to have good efficacy against grain pests without loss in grain quality while leaving almost no residue [6]. Phosphine is most often applied in the form of an aluminum phosphide. Aluminum phosphide, a 0.6 or 3 g grayish tablet, absorbs moisture from the air and decomposes into phosphine and aluminum dust. Released phosphine gas can easily spread inside grain piles within a short time [7]. Owing to its good penetration rate, low cost and convenience compared with those of other fumigants, aluminum phosphide is widely applied to control grain pests in most countries [8].

Although phosphine is a fumigant with many advantages, some unavoidable features, such as long exposure time, dust problems or considerable worker safety issues, interrupt the expanded use of phosphine in other fields [9,10]. Furthermore, many countries have recently reported the occurrence of phosphine-resistant pests [11]. Grain pests that have become resistant to phosphine are not easily killed under common phosphine treatment; therefore, fumigation must be performed at higher concentrations and for longer exposure times. In particular, it has been reported that complete control with phosphine is impossible in cases of strong resistance caused by genetic mutations in two loci [12,13]. To inhibit the spread of phosphine-resistant pests, several alternative fumigation methods have been developed. Methyl bromide is a widely used quarantine fumigant, and it shows good efficacy against several pests [5]. However, it has been designated an ozone-depleting substance by the Montreal Protocol and is being phased out [14]. Ethyl formate has been recently developed and commercialized as a methyl bromide alternative fumigant and is registered as a quarantine treatment method for imported fruits and vegetables in Korea [15,16]. Ethyl formate is a nature-derived material; thus, compared with methyl bromide, it is safer for both humans and the environment [17]. Ethyl formate treatment is effective at controlling several grain pests, yielding less damage to grain quality, and is even effective against phosphine-resistant pests [18,19]. However, its drawbacks, such as high sorption and flammability, should be overcome by combining it with other treatment methods [20]. Other fumigation materials, such as methyl benzoate, acetophenone or anisole, have been reported to have significant effects on grain pests, and research on commercializing these materials is currently in progress [21,22,23]. Carbonyl sulfide (COS) has also been newly developed as a fumigant against phosphine-resistant pests for durable commodities [24]. COS is a naturally occurring gas and has low acute inhalation toxicity [25]. It was newly patented by CSIRO Australia in 1992 as a new fumigant for grain, and first announced to the scientific community in 1994 [24]. Viljoen and Ren [26] reported that COS can penetrate easily into commodity compared to methyl bromide. Zettler et al. [27] reported that COS has good efficacy against grain pests; thus, it has potential for use as a fumigant for dried fruits and nuts. Some researchers reported that COS may be suitable as a fumigant for fresh fruits like avocado, papaya, and lemon; however, it can cause slight damage to fruits [28,29,30]. Lee et al. [31] reported that COS can control phosphine-resistant *T. castaneum* at a dosage similar to that used against phosphine-susceptible strains. This study investigated the efficacy of COS as a new fumigant on an important grain pest, *S. oryzae*, which can cause significant loss of stored grain. We performed a comparative evaluation of the efficacy of phosphine and COS on phosphine-resistant and phosphine-susceptible *S. oryzae* to verify whether COS can be applied for controlling phosphine-resistant pests.

## 2. Materials and Methods

### 2.1. Tested Insects

Phosphine-susceptible and phosphine-resistant strains of *S. oryzae* were obtained from Entomotoxicology lab, Chungbuk National University (Cheongju, Republic of Korea). Both strains were reared separately in the insect breeding room of the Animal and Plant Quarantine Agency (APQA). The rearing room was maintained at 25 ± 1 °C and 60 ± 10% relative humidity under 14:10 h (L:D) light conditions. Both strains of *S. oryzae* were fed organic rice regularly. Egg (1 d), larva (early), and adult (1 week after emergence) stages have been used for this experiment.

### 2.2. Fumigants

Phosphine fumigant (2% PH_3_ + 98% CO_2_) was purchased from FarmHannong (Seoul, Republic of Korea). COS (98%) was purchased from Aldrich Chemical Company, Inc. (St. Louis, MO, USA).

### 2.3. Testing the Efficacy of Phosphine and COS Against Sitophilus oryzae

All the fumigation tests were conducted at the Plant Quarantine Research Center in APQA (Gimcheon, Republic of Korea). Tested insects (30 individuals per replicate) of each growth stage were stored separately in 50.00 × 15.00 mm breeding dishes (SPL Life Science, Pocheon, Republic of Korea) with a small amount of food (unpolished organic rice) prior to the experiment, and fumigation was conducted with 12 L acrylic desiccators (UniB&C, Goyang, Republic of Korea) using methods similar to those described by Lee et al. [31]. Briefly, the fumigant gas was transferred from the cylinder to a 10 L Tedlar bag (SKC Inc., Eighty Four, PA, USA) and injected into a desiccator with a 0.5 L gas-tight syringe (Trajan Scientific and Medical, Ringwood, Victoria, Australia), after which the dose was calculated to 10.0, 15.0, 30.0, 35.0, 40.0, 45.0, 50.0 g/m^3^ (COS) and 0.0001, 0.001, 0.01, 0.1, 0.5, 1.0, 1.5, 2.0, 3.0, 4.0 g/m^3^ (phosphine), depending on the strain. Fumigation was performed for 24 h at room temperature (25 ± 1 °C). After fumigation, the desiccators were opened and ventilated for 1 h. All the breeding dishes with insects were moved to the insect rearing room, and the mortality at the adult and larval stages was determined by visual inspection under a microscope at 5 days after fumigation, considering the knock-down effects. The mortality at the egg and pupal stages was determined by counting the number of hatched eggs and emerged adults, respectively, for 2 weeks. The pupal stage was used for only COS analysis in this study because, in our previous study, phosphine tolerance between susceptible and resistant pupae of *S. oryzae* was not significantly different [32]. Corrected mortality was calculated using the formula [33]: corrected mortality = (% treatment mortality − % control mortality)/(100 − % control mortality) × 100. All the treatments were replicated three times.

### 2.4. Measurement of Fumigant Concentrations and Concentration Time (Ct)

To measure the concentration of fumigant in the desiccators, mixed gas inside the desiccator was sampled with a gas-tight syringe at 0, 1, 2, 4 and 24 h after exposure. Sampled gas was measured by gas chromatography (GC-8890, Agilent, Santa Clara, CA, USA) equipped with a flame photometric detector (FPD) and flame ionization detector (FID). The injector and oven temperatures were 200 °C, and the detector temperature was 200 °C. The injection amount of phosphine and COS was 10 μL, and the fumigant concentration was calculated on the basis of the peak area for the external standard.

The Ct value was calculated using the equation described by Monro [34] on the basis of the periodically monitored gas concentration.

Ct = ∑(C_i_ + C_i+1_) (t_i+1_ − t_i_)/2 (1)

where C is the fumigant concentration (mg/L), t is the time of exposure (h), i is the order of measurement, and Ct is the concentration × time (mg h/L).

### 2.5. Statistical Analysis

On the basis of the efficacy of fumigants against *S. oryzae*, the significance of the effects of the fumigant Ct value was determined using the SPSS 25 program (IBM corp., Armonk, NY, USA). The 50% and 99% lethal Ct (LCt_50_ and LCt_99_, respectively) values of the fumigant were obtained by using probit analysis [35]. The resistance ratio between the phosphine-susceptible strain and the phosphine-resistant strain was calculated on the basis of the methods of Jagadeesan and Nayak [36].

## 3. Results

### 3.1. Phosphine Efficacy Against Phosphine-Susceptible Sitophilus oryzae

At the larval stage, the mortality rate of phosphine-susceptible *S. oryzae* was 100% at the lower concentration, 0.1 mg/L, whereas at the egg stage, the mortality rate was 100% at the higher concentration, 1.0 mg/L. The results of the probit analysis revealed that the LCt_99_ values for the eggs, larvae and adults were 37.11, 16.70 and 15.37 mg h/L, respectively (Table 1).

### 3.2. Phosphine Efficacy Against Phosphine-Resistant Sitophilus oryzae

At the adult stage, the mortality rate of phosphine-resistant *S. oryzae* was 100% at the lowest concentration, 2.0 mg/L, while at the egg and larval stages, the mortality rate was 100% at concentrations greater than 3.0 mg/L. The results of the probit analysis revealed that the LCt_99_ values for the eggs, larvae and adults were 149.36, 84.77 and 50.93 mg h/L, respectively (Table 2).

### 3.3. COS Efficacy Against Phosphine-Susceptible Sitophilus oryzae

At the adult stage, the mortality rate of phosphine-susceptible *S. oryzae* was 100% at the lowest concentration, 30 mg/L, while the mortality rates at the egg and pupal stages were 100% at concentrations greater than 50 mg/L. The results of the probit analysis revealed that the LCt_99_ values for the eggs, larvae, pupae and adults were 940.55, 385.69, 1316.92, and 278.03 mg h/L, respectively, indicating that the pupal stage was the most tolerant stage among all the growth stages (Table 3).

### 3.4. COS Efficacy Against Phosphine-Resistant Sitophilus oryzae

Phosphine-resistant *S. oryzae* at the larval and adult stages exhibited 100% mortality rates at the lowest concentration, 35 mg/L, while that at the egg and pupal stages presented 100% mortality rates at concentrations greater than 50 mg/L. The results of the probit analysis revealed that the LCt_99_ values for the eggs, larvae, pupae, and adults were 994.78, 404.47, 1184.67, and 358.73 mg h/L, respectively, indicating that the pupal stage was the most tolerant stage among all the growth stages (Table 4).

### 3.5. Comparative Efficacy of Phosphine and COS Against Sitophilus oryzae with and Without Phosphine Resistance

The phosphine resistance ratios between the phosphine-susceptible and phosphine-resistant *S. oryzae* eggs, larvae, and adults were 21.28, 27.94 and 12.68 times, respectively (Table 5). However, the COS resistance ratios between the phosphine-susceptible and phosphine-resistant *S. oryzae* eggs, larvae and adults were 1.02, 0.88, 0.64, and 1.08 times, respectively, indicating that there were no differences in mortality between phosphine-susceptible and -resistant insects (Table 6).

## 4. Discussion

Recently, the increase in phosphine resistance has become among the most important problems in global agriculture. Therefore, many countries are using various strategies to reduce concerns regarding damage to stored grain [8,13]. As methyl bromide cannot be used on domestic crops according to Montreal protocols, most stored grains are fumigated with phosphine in Korea, and the risk of the emergence of phosphine-resistant pests is increasing. Yang et al. [37] first reported the occurrence of phosphine-resistant *S. oryzae* in Korea, and the phosphine dosage required to control the resistant strain was 10-fold greater than that required for the susceptible strain. Kim et al. [38] reported that five field-collected strains of *T. castaneum* presented no P45S point mutation in the dihydrolipoamide dehydrogenase (dld) gene, but one strain presented 1.7-fold greater gene expression than the susceptible strain did. Similar trends are emerging in other countries. Daglish et al. [39] reported that the resistance ratio for *S. oryzae* in Australia was 12-fold, and Behera et al. [40] reported 9.5-fold resistance from field-collected *S. oryzae* in India. Considering the current situation, with the ban on methyl bromide and the increase in phosphine resistance, the development of countermeasures against phosphine resistance is imperative [41].

In our study, the efficacy of phosphine against *S. oryzae* differed in terms of resistance, as phosphine-susceptible insects presented lower LCt_50_ values (0.63 to 1.44 mg h/L), whereas phosphine-resistant insects presented higher LCt_50_ values (8.37 to 30.65 mg h/L). The resistance ratio between phosphine-susceptible and phosphine-resistant insects ranged from 12.68- to 27.94-fold. These results indicate that more exposure time or a greater concentration of phosphine is required to control phosphine-resistant insects [42]. It is generally assumed that increasing the fumigation time is more important than increasing the dosage for controlling grain pests [43,44]. However, Daglish [45] reported that dosage and time contribute similarly to the mortality of *S. oryzae* adults (toxicity index ‘n’ = 1.0073). These results suggest that a more complex approach is needed to control *S. oryzae* beyond extending the treatment period, which is the conventional approach. Since we used early larvae in this study, the LCt_50_ values were similar to those obtained in our previous research [32]. However, unlike our previous findings, the egg stage appeared to be the most tolerant growth stage, although the broad CL ranges make it difficult to confirm. This is assumed to be due to differences in the growth time of the insects used in each experiment. Nakakita and Winks [46] reported that the LC_50_ value for *T. castaneum* can differ according to the immature stage and that the LC_50_ value for late pupae is similar to that of the larval stage, while early pupae are 32 times more tolerant than larvae, suggesting that the efficacy can differ across growth stages and even at the same stage. Therefore, further research is needed to determine whether the effects of concentration and time vary depending on the growth time of the insect.

In contrast, COS showed reliable efficacy against both strains of *S. oryzae*, regardless of phosphine resistance. When treated with COS, phosphine-susceptible insects presented LCt_50_ values ranging from 171.11 to 284.19 mg h/L, and phosphine-resistant insects presented LCt_50_ values ranging from 149.87 to 289.78 mg h/L. The resistance ratio between phosphine-susceptible and phosphine-resistant insects ranged from 0.88- to 1.08-fold, indicating that COS shows no cross-resistance to phosphine [36]. Lee et al. [31] reported the same results: Both phosphine-susceptible and phosphine-resistant *T. castaneum* strains showed similar efficacy when treated with COS. This is because COS has a different mode of action than phosphine does. Shin et al. [47] reported that phosphine-susceptible and phosphine-resistant *T. castaneum* exhibit similar mortality rates when treated with COS, and this is because although disruption of mitochondrial function is commonly involved in the mechanism of action of phosphine and COS, the enzyme expression patterns differ. Ethyl formate and sulfuryl fluoride, promising phosphine alternative fumigants, are also known to have no cross-resistance and have different modes of action than those of phosphine [36,48]. Although there were no differences in mortality at any growth stage between phosphine-resistant and phosphine-susceptible *S. oryzae* when they were treated with COS, the efficacy differed across insect growth stages. Compared with that at the larval and adult stages, *S. oryzae* at the egg stage of was more tolerant to COS treatment, and this difference appeared in similar patterns in both phosphine-resistant and phosphine-susceptible insects. Lee et al. [31] reported that the mortality of phosphine-resistant and phosphine-susceptible *T. castaneum* was 100% when they were treated with 40.23 mg/L COS, and compared with that at the larval and adult stages, *T. castaneum* at the egg and pupa stages was more tolerant. *T. castaneum* at the pupa and egg stages is known to be more tolerant to fumigants because most fumigants take effect through respiration [49,50,51]. Therefore, the treatment level of COS should be set on the basis of the tolerant growth stages, that is, the eggs and pupae.

Currently, the phosphine resistance problem in South Korea is not considered serious compared with that in other countries [52]. Although it is currently manageable, if not controlled urgently, it could become more serious in the future. Emery et al. [53] reported a 20-year resistance frequency in Australia, and a steady increase in weak resistance and a similar build-up in strong resistance have been observed. Pimentel et al. [54] reported that the resistance ratio for field-collected *Sitophilus zeamais* in Brazil was less than 10-fold in most areas, but the resistance ratio has been reported to be 86.58-fold in some areas. Agrafioti et al. [55] reported that among field-collected *S. oryzae*, 4 out of 6 regions were confirmed to be phosphine resistant, and more than 80% of the pests survived in 2 regions after the Food and Agriculture Organization (FAO) protocol treatment. In this study, COS showed reliable efficacy in controlling phosphine-resistant *S. oryzae*, indicating that COS can be used as an alternative fumigant to phosphine for plant quarantine and stored grain pest control purposes. However, more studies should apply COS in the field. Ethyl formate, developed prior to the use of COS as a phosphine alternative fumigant, has demonstrated high efficacy against phosphine-resistant pests, similar to that of COS [56,57,58]. However, the high initial concentration, due to high sorption, incurs increased costs, thereby hindering field application. To address this issue, several researchers have reported combined treatment with ethyl formate and phosphine or physical treatment to reduce fumigant usage [59,60,61,62]. If these studies are also conducted with COS, it is expected that COS will contribute to reducing the phosphine-resistance problem.

## Figures and Tables

**Table 1:** Lethal concentration time values for phosphine-susceptible *S. oryzae* exposed to phosphine.

Stage	*n*	LCt_50_ (mg h/L) (95% CL ^a^)	LCt_99_ (mg h/L) (95% CL)	Slope ± SE ^b^	*df*	χ^2^
Egg	630	1.44 (0.76–2.57)	37.11 (21.57–177.23)	1.65 ± 0.20	8	0.97
Larva	630	0.63 (0.18–1.29)	16.70 (2.70–123.18)	1.61 ± 0.30	8	0.75
Adult	630	0.66 (0.13–1.44)	15.37 (7.01–186.33)	1.74 ± 0.23	6	0.69

^a^ CL denotes the confidence limit. ^b^ SE denotes the standard error.

**Table 2:** Lethal concentration time values for phosphine-resistant *S. oryzae* exposed to phosphine.

Stage	*n*	LCt_50_ (mg h/L) (95% CL ^a^)	LCt_99_ (mg h/L) (95% CL)	Slope ± SE ^b^	*df*	χ^2^
Egg	630	30.65 (14.31–54.41)	149.36 (89.12–726.85)	3.20 ± 0.79	8	0.59
Larva	630	17.60 (12.69–24.41)	84.77 (68.02–153.03)	1.76 ± 3.41	8	0.78
Adult	630	8.37 (2.22–17.37)	50.93 (37.85–148.87)	2.97 ± 0.52	6	0.86

^a^ CL denotes the confidence limit. ^b^ SE denotes the standard error.

**Table 3:** Lethal concentration time values for phosphine-susceptible *S. oryzae* exposed to COS.

Stages	*n*	LCt_50_ (mg h/L) (95% CL ^a^)	LCt_99_ (mg h/L) (95% CL)	Slope ± SE ^b^	*df*	χ^2^
Egg	720	284.19 (206.25–349.01)	940.55 (674.17–2039.28)	4.47 ± 0.36	6	2.19
Larva	720	171.11 (146.08–187.95)	385.69 (325.64–540.54)	3.02 ± 0.28	6	1.22
Adult	720	212.55 (198.65–261.64)	278.03 (247.95–345.96)	2.00 ± 0.24	6	3.45

^a^ CL denotes the confidence limit. ^b^ SE denotes the standard error.

**Table 4:** Lethal concentration time values for phosphine-resistant *S. oryzae* exposed to COS.

Stage	*n*	LCt_50_ (mg h/L) (95% CL ^a^)	LCt_99_ (mg h/L) (95% CL)	Slope ± SE ^b^	*df*	χ^2^
Egg	720	289.78 (201.90–380.81)	994.78 (784.20–1471.60)	4.34 ± 0.33	6	5.33
Larva	720	149.87 (98.14–187.83)	404.47 (267.27–788.47)	5.40 ± 0.33	6	5.12
Adult	720	229.06 (188.21–298.30)	358.73 (271.14–642.83)	6.94 ± 0.78	6	4.17

^a^ CL denotes the confidence limit. ^b^ SE denotes the standard error.

**Table 5:** Phosphine resistance ratio for phosphine-susceptible and phosphine-resistant *S. oryzae*.

Stage	LCt_50_ Value for Phosphine-Susceptible *S. oryzae* (mg h/L)	LCt_50_ Value for Phosphine-Resistant *S. oryzae* (mg h/L)	Resistance Ratio ^a^
Egg	1.44 (0.76–2.57)	30.65 (14.31–54.41)	21.28
Larva	0.63 (0.18–1.29)	17.60 (12.69–24.41)	27.94
Adult	0.66 (0.13–1.44)	8.37 (2.22–17.37)	12.68

^a^ Resistance ratio = (LCt_50_ for the resistant strain/LCt_50_ for the susceptible strain).

**Table 6:** COS resistance ratio for phosphine-susceptible and phosphine-resistant *S. oryzae*.

Stage	LCt_50_ Value for Phosphine-Susceptible *S. oryzae* (mg h/L)	LCt_50_ Value for Phosphine-Resistant *S. oryzae* (mg h/L)	Resistance Ratio ^a^
Egg	284.19 (206.25–349.01)	289.78 (201.90–380.81)	1.02
Larva	171.11 (146.08–187.95)	149.87 (98.14–187.83)	0.88
Adult	212.55 (198.65–261.64)	229.06 (188.21–298.30)	1.08

^a^ Resistance ratio = (LCt_50_ for the resistant strain/LCt_50_ for the susceptible strain).

## Data Availability

The original contributions presented in this study are included in the article. Further inquiries can be directed to the corresponding author.
